# Fear of COVID-19, prolonged smartphone use, sleep disturbances, and depression in the time of COVID-19: A nation-wide survey

**DOI:** 10.3389/fpsyt.2022.971800

**Published:** 2022-10-14

**Authors:** Gangqin Li, Hao Liu, Changjian Qiu, Wanjie Tang

**Affiliations:** ^1^Department of Forensic Psychiatry, West China School of Basic Medical Sciences and Forensic Medicine, Sichuan University, Chengdu, China; ^2^West China School of Basic Medical Sciences and Forensic Medicine, Sichuan University, Chengdu, China; ^3^Mental Health Centre, West China Hospital, Sichuan University, Chengdu, China

**Keywords:** COVID-19, depression, smartphone use, pandemic fear, sleep disturbance

## Abstract

**Background:**

The COVID-19 pandemic has had a wide range of behavioral and psychological effects on the general population. This study examined the relationship between fear of COVID-19, daily smartphone use, sleep disturbance, and depression in the general population during the early stage of COVID-19.

**Methods:**

An online nation-wide survey was conducted from March 20 to April 10, 2020. Sociodemographic information, including age, gender, educational attainment, vocation, and duration of self-isolation, was collected; fear of COVID-19 and other objective exposures, daily hours of smartphone use, night sleep duration, sleep disturbance, and depressive symptoms were measured with structured questions and PHQ-9. There were 1,280 questionnaires in total, and 1,250 valid questionnaires remained.

**Results:**

The prevalence of sleep disturbance and depression were found to be 13.1 and 10.7%, respectively. Feelings of extreme fear, longer smartphone use, difficulty initiating sleep, and early morning awakening were significant risk factors for depression. Daily hours of smartphone use, difficulty initiating sleep, and early morning awakening partially mediated the association between feeling extremely scared of the pandemic and depression.

**Conclusion:**

Psychological interventions in a major public health crisis should focus more on the subjective perception of pandemic fear. At the same time, daily smartphone use and sleep disturbances could serve as targets for monitoring and intervention for depression during a pandemic.

## Introduction

The coronavirus disease (COVID-19) emerged in late 2019, and has caused widespread societal disruption, morbidity, and loss of life globally (1–3). To curb the spread of the virus, many countries implemented quarantine measures, such as lockdowns, home isolation, and social distancing (4). The pandemic and the quarantine significantly changed people's survival environments, behaviors, and lifestyles, affecting their psychological wellbeing and resulting in increased mental health problems (5–10).

The COVID-19 pandemic has been reported to be a traumatic stressor that can cause posttraumatic stress disorder (PTSD)-like responses (11–13). Furthermore, fear of infection can also result in various psychological disorders (14), such as anxiety, sleep disturbances, and depressive symptoms (8, 15–19). On the other hand, the secondary negative consequences of the pandemic, such as unemployment, economic adversity, and fewer social and communication opportunities, may also result in many negative feelings, such as boredom, loneliness, anxiety, depression, and even suicidality (2, 3, 5, 20, 21). Among these negative psychological consequences, depression is one of the most commonly reported and addressed. Several previous studies have reported higher rates of depression during the COVID-19 pandemic. For example, Jones and collogues reviewed studies in 2019–2021 and found that global adolescents experienced higher rates of depression due to the pandemic (22). The COVID-19 Mental Disorders Collaborators in Australia conducted a systematic review (23). They estimated there were 53.2 million cases of major depressive disorder globally (an increase of 27.6%) due to the COVID-19 pandemic, which caused 49.4 million DALYs globally in 2020. Depression, characterized by depressive mood (in adolescents, it may be described as an irritable or empty feeling), has various other symptoms, such as diminished interest, loss of pleasure, feelings of hopelessness and worthlessness, changes in appetite or sleep, and reduced energy or fatigue, or even suicidal ideation and attempts (24–26). Despite the high rates and vast influence of depression, the risk factors and pathological mechanisms remain largely unknown. Many researchers have called for special attention and more studies on the short- or long-term depressive symptoms due to the COVID-19 pandemic (27–31).

In the COVID-19 crisis, a significant increase in smartphone use and addiction has been observed by many researchers (32–34). For example, Zhang et al. found an elevated risk of problematic smartphone use among Chinese adults during the COVID-19 pandemic; the prevalence was as high as 43.3% (35). Another study reported that the prevalence of smartphone addiction among 6,154 undergraduates was up to 62.4% during the COVID-19 quarantine (36). Caponnetto and Serra also documented the growth of pathological use of the smartphone during the Italian lockdown caused by COVID-19 (37, 38). Proper smartphone use is beneficial but problematic, while addictive use is usually associated with a range of physical and mental health problems, such as dry eyes (39), migraine headaches (40), social withdrawal (41), sleep disorders (42), and depression (43–45).

As mentioned above, problematic smartphone use was associated with sleep disturbance and depression during the COVID-19 lockdown. Moreover, Smith et al. found a positive association between screen time per day in hours and poor mental health during COVID-19 self-isolation in a sample of four major countries (46). A persistent impact of the COVID-19 pandemic on sleep and mental health has also been found in Italy (47), and smartphone overuse predicted a higher risk of poor sleep and insomnia symptoms during the COVID-19 outbreak. Further, the bidirectional relationship between sleep disturbance and depression has also been well-established in previous studies (48, 49). Therefore, it is reasonable to presume that there may be a relationship between the three factors contributing to depression. However, most studies only examined the correlations between two of the three factors, while only a few investigated the association of the three factors simultaneously. Furthermore, the results varied between studies. For example, Perkinson-Gloor et al. reported that sleep disturbance partially mediated the relationship between electronic media use in bed before sleep and symptoms of depression in adolescents (50); this partial mediation role of sleep disturbance was also verified by a Chinese study based on college students (51). Huang et al. reported that poor sleep quality was significantly associated with 5 h of daily smartphone use and more severe depressive symptoms in Chinese college students (52). However, Taura et al. found that overall smartphone use of over 5 h per day was associated with shorter sleep duration and sleep disturbance, but not depression in Japanese adolescents (53). Cui et al. found that problematic smartphone use predicted subsequent sleep quality, and bedtime procrastination predicted subsequent depressive symptoms in Chinese college students (54).

On the contrary, Kang et al. found bidirectional longitudinal relationships between smartphone addiction and sleep quality; however, they did not find a predictive effect of smartphone addiction on depressive symptoms in a sample of Chinese college students (55). This inconsistency may be due to the differences in the sample, such as the majority of the sample being young adult college students, some being samples of teenagers, and a few being in other older age groups. Therefore, an age-wide representative sample of adults is needed to increase our understanding of the relationship between smartphone overuse, sleep disturbance, and depression.

Based on the above literature, we believe people might have overused their smartphones to relieve stress during the first wave of COVID-19, which could subsequently affect their sleep quality, resulting in serious sleep problems and even emotional problems. According to the general strain theory framework (56), when facing chronic, uncontrollable stress, people may develop maladaptive patterns to escape from negative emotions, such as extreme fear, which can have adverse physical and mental effects. For example, fear of COVID-19 could influence the general population's mental health *via* smartphone addiction (57). In addition, sleep disturbance tends to mainly include three different aspects, difficulty initiating sleep (DIS), difficulty maintaining sleep (DMS), and early morning awakening (EMA) (58). However, few studies have explored the relationship between these three dimensions of sleep disturbance and pandemic fear, smartphone overuse, and depression.

The present study sought to examine smartphone use, sleep disturbance dimensions, and depressive symptoms in a nation-wide general population at the early stage of COVID-19 in China. The associations between these three factors and the influencing factors of depression will be analyzed. Moreover, chain mediation analysis would also be used to investigate the potential mediating role of prolonged smartphone use and sleep disturbance dimensions in the relationship between pandemic fear and depression.

Based on previous research, it was hypothesized that:

H1: Feeling extremely scared of COVID-19 is associated with prolonged smartphone use, sleep disturbance dimensions (DIS, DMS, and EMA), and depression.

H2: Prolonged smartphone use mediates the relationship between feeling extremely scared of COVID-19 and depression.

H3: Sleep disturbance dimensions (DIS, DMS, and EMA) mediate the relationship between feeling extremely scared of COVID-19 and depression.

## Methods

### Study design and participants

This nation-wide online survey was sent out through WeChat moments and WeChat groups across the country in a snowball way from March 20 to April 10, 2020. This was just 2 months after the COVID-19 outbreak, and the strictest nation-wide lockdown was enforced (China enforced the first and most strict national lockdown on January 23, 2020). The new cases of COVID-19 decreased dramatically in late February, and the quarantine policies in China have been easing gradually since March). Each person to complete the questionnaire was awarded a random prize of 1 to 10 CNY to encourage greater participation. We included participants between the ages of 18 and 75 living in China. In total, 1,280 participants completed the survey, among whom 30 were excluded because they were < 18 years old; therefore, 1,250 participants were included in this study. It is noteworthy that, because it refers to a period with important differences in the restraining measures in force, the data collection on pandemic fear, sleep variables, and smartphone use in this study is purely exploratory.

This project was approved by the Ethics Committee of the Sichuan Psychological Society (2020_12), which was a part of the Survey on the Behavior and Psychological Health Project affected by COVID-19 (SBPHP_COVID-19) to assess the psychological impact of the pandemic outbreak on the general population. All questionnaires were anonymous, and informed consent was given by all participants on the first page of the questionnaire.

### Measurements

#### Demographic data and COVID-19 fear

Participants provided information on their gender, age, education level, vocation, income decrease, and home isolation duration due to the pandemic. COVID-19 fear was evaluated by one subjective question with a response of yes/no: whether they felt extremely scared since the pandemic outbreak. Objective exposure variables were also asked: whether a friend or relative had been infected with COVID-19; whether they lived in a community where someone was infected; and whether they lived in the worst-hit areas (at that time it included three regions or cities, i.e., Hubei Province, Guangzhou City, and Dalian City) (59).

#### Duration of smartphone use

The average smartphone use duration per day since the outbreak was determined with the following question: “How many hours have you been spending on your smartphone each day on average since the COID-19 outbreak?”

#### Sleep disturbance and sleep duration

Sleep disturbance since the outbreak (just about the last 2–3 months) was measured with three questions. The following three not-validated questions measured three aspects of sleep disturbance (60): (1) “Did you have difficulty initiating sleep within 30 minutes at night?” (DIS); (2) “Did you have difficulty maintaining sleep (DMS), such as waking during the night and having difficulty returning to sleep?”; (3) “Did you have any early morning awakening and have difficulty returning to sleep? (EMA).” The participants were required to write down how many nights, on average, per week they had these symptoms. The answers were then divided into “no,” “less than once a week,” “once or twice a week,” or “three or more times a week.” In addition, actual sleep duration per night on average since the outbreak was assessed with a single question: “Since the outbreak, how many hours of actual sleep per night, on average, did you have?”. The response categories were: < 5 h per night, 5–6 h per night, 6–7 h per night, 7–8 h per night, more than 9 h per night.

#### The Patient Health Questionnaire-9

Depressive symptoms in the past month were measured with the Patient Health Questionnaire-9 (PHQ-9) (54), a 9-item brief questionnaire for screening depression. This scale has been validated in the Chinese adult population and has been shown to have good psychometric properties (61). The total score of PHQ-9 ranges from 0 to 27, with a cut-off score of ≥10 indicating depression (62). In the current study, Cronbach's α was 0.844.

### Statistical analysis

The statistical analyses were performed using IBM SPSS Statistics Version 15 (IBM, Armonk, NY), with the significance level set at 0.05 (two-sided). The means and standard deviations were computed to describe the continuous variables, and frequencies were used to describe the categorical variables. The Chi-squared test was used to examine the differences in depression prevalence and other frequency data in different subgroups, while Bonferroni was used to correct multiple comparisons. Two-independent sample *t*-tests were used to explore the differences in PHQ scores in different subgroups. Cramer V was used to describe the association between nominal categorical data, while Kendall Tau-C was used to describe the association between ordinal categorical data. Binary logistic regression (Forward: LR for inclusion of variables) was applied to determine the risk factors for depression. Independent variables included age, gender, educational level, vocation, isolation time, income decrease, exposure severity to COVID-19, duration of smartphone use, night sleep duration, and sleep disturbance parameters. In contrast, the dependent variables were depression or non-depression. The PROCESS plug-in was applied to examine the pathways between variables from exposure to depression. The indirect effects and 95% bootstrap confidence intervals (CI) were calculated based on 5,000 bootstrapped samples.

## Results

### Sample characteristics

The mean age of the participants was 39.4 (SD = 10.3), 36.3% were male, 77.9% experienced consecutive home isolation for more than 14 days, 40.1% reported a decrease in income, 5.2% were non-community medical health workers, and 11.9% were community health staff. Approximately 21.0% reported feeling extremely scared. The objective COVID-19 exposure was as follows: 1.5% reported infected persons in their communities, 6.2% reported living in the worst-hit community, and 4.5% reported that a friend or relative was infected. Details are presented in the first two columns in Table 1.

**Table 1:** Demographic characteristics and depression prevalence in subgroups.

**Variables**	***N* (%)**	**Depression *N* (%)**	**χ^2^**	***P***	**Cramer V V/Tau C**
Total	1,250 (100.0)	134 (10.7)			
**Age**
18–35	450 (36.0)	56 (12.4)	3.306	0.219	0.049
36–55	749 (59.9)	75 (10.0)			
56–72	51 (4.1)	3 (5.9)			
**Gender**
Male	454 (36.3)	44 (9.7)	0.788	0.375	0.025
Female	796 (63.7)	90 (11.3)			
**Vocation**
Health workers	65 (5.2)	4 (6.2)^a^	6.193	0.045	0.070
Community health staff	149 (11.9)	24 (16.1)^a^			
Other	1,036 (82.9)	106 (10.2)^a^			
**Home isolated (>2 weeks)**
Yes	974 (77.9)	97 (10.0)	2.670	0.122	0.046
No	276 (22.1)	37 (13.4)			
**Income decrease**
Yes	501 (40.1)	63 (12.6)	3.006	0.093	0.049
No	749 (59.9)	71 (9.5)			
**Educational level**
Junior high school or below	254 (20.3)	26 (10.2)	4.237	0.375	0.058
Senior High school	335 (26.8)	37 (11.0)			
Junior college	277 (22.2)	22 (7.9)			
Bachelor degree	344 (27.5)	43 (12.5)			
Master degree or above	40 (3.2)	6 (15.0)			
**Subjective exposure**
Felt extremely scared
Yes	262 (21.0)	68 (26.0)	80.379	< 0.001	0.254
No	1,172 (79.0)	66 (6.7)			
**Objective exposure**
**Community infected**
Yes	19 (1.5)	7 (36.8)	13.755	0.002	0.105
No	1,231 (98.5)	127 (10.3)			
**Friend or relative infected**
Yes	56 (4.5)	14 (25.0)	12.491	0.001	0.100
No	1,194 (95.5)	120 (10.1)			
**Living in the worst-hit areas**
Yes	78 (6.2)	19 (24.4)	16.169	< 0.001	0.114
No	1,172 (93.8)	115 (9.8)			

The *p*-value is for the difference in depression prevalence in different subgroups under the titled demographic and exposure category (for example, subgroups in age, gender, vocation, and so on). The letter “^a^” denotes that the column proportions do not differ significantly at the 0.05 level.

### Depression prevalence in different subgroups

Comparisons of the prevalence of depression in different subgroups are presented in Table 1. Of the participants, 10.7% (*N* = 134) were categorized as having depression based on the cut-off score of 10 on PHQ-9. It can be seen that the prevalence of depression only differed in participants with different levels of exposure to COVID-19; those with higher severity of exposure to the pandemic had a higher rate of depression. Besides, no differences in depression prevalence were found in subgroups with different genders, ages, vocations, educational level, income decline, or duration of isolation.

### Duration of smartphone use in different subgroups

Comparisons of the duration of smartphone use in different subgroups are presented in Supplementary Table S1. Younger participants (18–35 years old), males, or participants with income decreased due to the pandemic had longer smartphone use. Meanwhile, participants who lived in the worst-hit community or felt extremely scared had longer smartphone use. Participants with senior high school educational levels spent the least time on smartphones. Health worker participants spent less time on smartphone activities than participants with other vocations.

### Night sleep duration in different subgroups

Sleep duration per night in different subgroups is presented in Supplementary Table S2. Younger participants (18–35 years old) had longer nights' sleep than elderly participants. Participants who experienced more than 14 consecutive days of home isolation had a longer night of sleep. On the contrary, community health staff had a shorter sleep duration than non-community health workers and participants with other vocations. Participants who lived in a community with infected persons or felt extremely scared had a shorter night's sleep duration than those without these conditions.

### Interactions between smartphone use duration, night sleep duration, and depression

Table 2 shows the sleep features and their associations with depression. The overall prevalence of sleep disturbance is 13.1%: difficulty initiating sleep (6.3%), difficulty maintaining sleep (9.7%), and early morning awakening (5.9%). Approximately 7.4% reported sleeping < 5 h, and 10.0% reported sleeping more than 9 h per night. Participants with sleep disturbances had a significantly higher rate of depression. The relationship between night sleep duration and the prevalence of depression formed a *U*-shaped curve. Participants with 8–9 h of sleep per night had the lowest rate (4.6%) of depression, while the rates increased as the sleep duration increased or decreased, and subgroups with five or fewer hours of sleep duration per night had the highest rate (28.3%) of depression. The frequencies of difficulty initiating sleep, maintaining sleep, and early morning awakening were all positively correlated with the prevalence of depression.

**Table 2:** Sleep features in the general population and depression prevalence in subgroups with different sleep features.

**Variables**	**Total *N* (%)**	**Depression *N* (%)**	**χ^2^**	**Adjusted *P***	**Cramer V/Tau-c**
Total	1,250 (100.0)	134 (10.7)			
**Sleep duration, hour**
≦5	92 (7.4)	26 (28.3)^a^	51.521	< 0.001	0.203
(5,6)*	230 (18.4)	37 (16.1)^a, b^			
(6,7)	247 (19.8)	24 (9.7)^b, c^			
(7,8)	426 (34.1)	26 (6.1)^b^			
(8,9)	130 (10.4)	6 (4.6)^b^			
>9	125 (10.0)	15 (12.0)^b, c^			
**Difficulty initiating sleep**
≥3 nights/wk	79 (6.3)	40 (50.6)^a^	240.200	< 0.001	0.438
1–2 nights/wk	113 (9.0)	36 (31.9)^a^			
< 1 night/wk	201 (16.1)	31 (15.4)^b^			
No	857 (68.6)	27 (3.2)^c^			
**Difficulty maintaining sleep**
≥3 nights/wk	74 (5.9)	29 (39.2)^a^	105.727	< 0.001	0.291
1–2 nights/wk	115 (9.2)	27 (23.5)^a, b^			
< 1 night/wk	213 (17.1)	29 (13.6)^b^			
No	848 (37.8)	49 (5.8)^c^			
**Early morning awakening**
≥ nights/wk	121 (9.7)	47 (38.8)^a^	163.191	< 0.001	0.361
1–2 nights/wk	141 (11.3)	34 (24.1)^a^			
< 1 night/wk	254 (20.3)	25 (9.8)^b^			
No	734 (58.7)	28 (3.8)^c^			
**Overall insomnia**
Yes	164 (13.1)	62 (37.8)	144.687	< 0.001	0.340
No	1,086 (86.9)	72 (6.6)			

The *p*-value is for the difference in depression prevalence in different subgroups under the titled sleeping disturbance category (for example, subgroups in sleep duration, difficulty initiating sleep, and so on). The letter “^a^,” ^“b^,″ or ^“c^″ denotes the column proportion does not differ significantly from those with the same superscripts at the 0.05 level but differs significantly from those with different superscripts at the 0.05 level. ^*^The number near the round bracket is not included in the dataset, while the number near the square bracket is included in the dataset.

**Figure 1:**
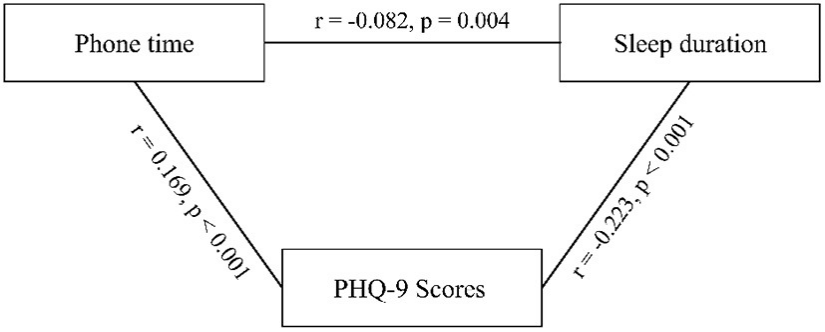
Partial correlations between Phonetime, Sleep duration and PHQ-9 scores after controlling for the age, gender, education level, income decrease.

Table 3 shows the association between smartphone use duration and depression. Clearly, smartphone use duration per day was positively associated with depression rate, and participants with five or more hours of smartphone use per day had the highest rate of depression, while participants with < 5 h of smartphone use per day did not differ in depression prevalence (see Table 3). Figure 1 shows the partial correlations between smartphone use duration, night sleep duration, and depression when controlling for gender, vocation, and income decline.

**Table 3:** Prevalence of depression in subgroups with different smartphone use duration.

**Smartphone use, hours**	**Total *N* (%)**	**Depression *N* (%)**	**χ^2^**	**Adjusted *P***	**Claimer V**
1	68 (5.4)	7 (10.3)^b^	27.822	< 0.001	0.149
2	216 (17.4)	21 (9.7)^b^	–	–	–
3	442 (35.4)	28 (6.3)^b^	–	–	–
4	261 (20.9)	27 (10.3)^b^	–	–	–
≥5	263 (21.0)	51 (19.4)^a^	–	–	–

*P*-value is for the difference in depression prevalence in subgroups with different daily hours of smartphone use. The letter “^a^” or “^b^” denotes the column proportion that does not differ significantly from those with the same superscripts at the 0.05 level but differs significantly from those with different superscripts at the 0.05 level.

### Binary logistic regression analysis

Table 4 shows the results of the binary logistic regression analysis. The dependent variable is depression or non-depression, and the independent variables are demographic factors, exposure to the pandemic, duration of smartphone use, and sleep disturbance. The most significant variable in the binary regression model for depression was DIS (≥3 nights/week) (OR = 10.504), DIS (1–2 nights/week) (OR = 5.658), and DIS (< 1 night/week) (OR = 3.833) relative to no DIS symptoms. The ORs for early morning awakening were higher for ≥3 nights/week (OR = 2.703), 1–2 nights/week (OR = 2.655) compared to no such symptom. Other significant variables in the models for depression were: prolonged use of a smartphone (OR = 1.245), people infected in their community (OR = 4.305), and feeling extremely scared (OR = 2.797).

**Table 4:** Variables associated with depression based on the binary logistic regression.

**Variables**	**Depression**	***P***
	**Adjusted OR (95% CI)**	
**Difficulty initiating sleep**
**No**	1.00	
< 1 night/week	3.833 (2.063–7.120)	< 0.001
1–2 nights/week	5.658 (2.901–11.036)	< 0.001
≥3 nights/week	10.504 (4.726–23.347)	< 0.001
**Early morning awakening**
**No**	1.00	
< 1 night/week	1.567 (0.804–3.054)	0.187
1–2 nights/week	2.655 (1.308–5.387)	0.007
≥3 nights/week	2.703 (1.188–6.147)	0.018
Smartphone use duration	1.245 (1.088–1.425)	0.001
**Objective exposure**
**People infected in their community**
No	1.00	
Yes	4.305 (1.131–16.386)	< 0.001
**Felt extremely scared**
No	1.00	
Yes	2.797 (1.775–4.409)	< 0.001

### Mediation analysis

**Figure 2:**
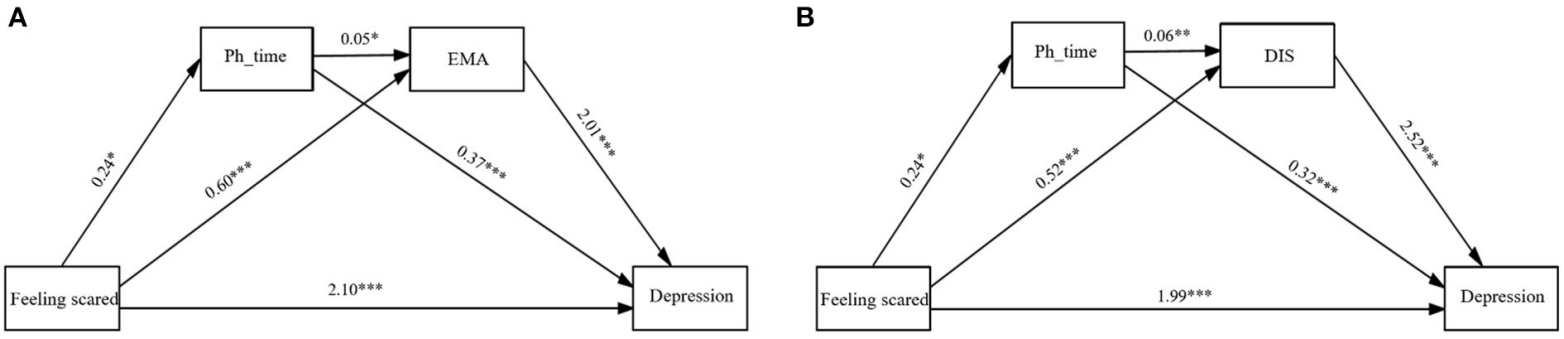
Path diagram between exposure, smartphone use duration, early morning awakening, **(A)** difficult initiating sleep **(B)** and depression. Ph_time, smartphone use duration; DIS, difficult initiating sleep; EMA, early morning awakening.

After controlling for age and gender, mediation analysis showed that the total effect of feeling extremely scared on depression was 3.419 (SE = 0.370, 95% CI = 2.693–4.144), in which direct effect accounted for about 70% of the total effect. Indirect effects accounted for about 30% through prolonged smartphone use and DIS/ EMA. Feeling extremely scared of COVID-19 had indirect effects on depression through smartphone use duration (β = 0.077, SE = 0.039, 95% CI = 0.018–0.173), DIS (β = 1.317, SE = 0.202, 95% CI = 0.946–1.733), and EMA (β = 1.209, SE = 0.181, 95% CI = 0.876–1.591), respectively. Further, mediation paths of exposure → smartphone use duration → DIS → depression (β = 0.036, SE = 0.020, 95% CI = 0.008–0.089) and exposure → smartphone use duration → EMA → depression (β = 0.023, SE = 0.015, 95% CI = 0.003–0.065) was also verified by the mediation analyses. Path diagrams are presented in Figure 2, and detailed data are in Supplementary Table S3.

## Discussion

The current study showed that 10.7% of the participants had depression. This rate is similar to that of 12.4% reported in Italy's large-scale survey conducted in March and May 2020 (63). Younger participants (18–35 years old), males, or participants with income decreased due to the pandemic had longer smartphone use, which indicates more attention should be drawn to these residents. Community health staff and participants who lived in a community with infected persons or felt extremely scared had shorter night's sleep duration than those without these conditions. These two kinds of people may face higher psychological pressure, influencing their sleep. Since sleep disturbance is associated with various physical and mental health problems, it should be a critical target for psychological intervention in these two kinds of residents.

In the current study, feeling extremely scared of the pandemic was found to be a significant risk factor for depression, suggesting that the subjective perception of the pandemic contributes more to subsequent mental distress. Several previous studies confirmed this finding. For example, Ding et al. have reported that affective risk perception is positively associated with depression in people in the COVID-19 crisis. In contrast, a distanced perception of public health crises was negatively associated with depression (64). Lakhan et al. noted that the fear of contracting the virus, lack of treatment, higher mortality associated with the virus, and uncertainty about when the virus would be controlled are the major factors that were found to be highly responsible for increasing depression and other mental health problems during the COVID-19 pandemic (65). Tsang et al. also reported that pandemic fear mediated the association between COVID-19 exposure and depression (66). These findings indicate that crisis psychological intervention should pay more attention to individuals' subjective feelings, and feeling extremely scared should be considered a risk factor for mental problems in the context of a pandemic.

Path analysis identified three indirect pathways from feeling scared of COVID-19 to depression. The first kind of pathway is feeling extremely scared → prolonged smartphone use → depression, indicating that feeling extremely scared could be associated with depression through the mediating role of prolonged smartphone use. Participants feeling extremely scared may hope to get as much information about the pandemic as possible or seek alleviation of their fear and distress through online activities, such as watching TV, gaming, shopping, and communicating through smartphones (67–69). Volpe et al. observed a rise in problematic internet use, social media addiction, and problematic video gaming during COVID-19. Pandemic-related general psychopathology and stress play a significant role in this behavior (70). However, excessive or addictive use is usually associated with a range of physical and mental health problems, such as dry eyes (39), migraine headaches (40), social withdrawal (41), sleep disorders (42), anxiety, and depression (43–45). The current study found that people who spent five or more hours per day on smartphone activities had significantly higher rates of depression. This result was in line with a previous study reporting that adolescents' prolonged smartphone use of ≥5 h on the weekend was associated with an increased risk of depressive symptoms (71). A prospective cohort study re-reported that smartphone use of ≥ 4 h/day could predict mental health problems in a college student sample (45, 72–74). The possible mechanism underlying this relationship remains to be clarified. One possible explanation is that prolonged smartphone use reduces face-to-face communication time with family members or friends, which could lead to functional impairment, interpersonal relationship problems, and tremendous difficulties in withdrawal (75, 76). Another explanation is that prolonged smartphone use increases fatigue, pain, irritability, and even cognitive impairments, resulting in depressive symptoms (77, 78).

The second kind of pathway identified is feeling extremely scared → smartphone use duration → DIS/EMA → depression. This was in line with several studies that reported that electronic media use was related to sleep difficulties in adolescents (79) and adults (80). For example, previous studies have found that the smartphone's bright screen light may suppress melatonin secretion and change the sleep structure, for instance, delaying sleep onset (81). Thomée et al. have noted that electronic media use may increase mental, emotional, or physiological arousal, resulting in sleep disturbance (e.g., longer sleep latency) (82). In addition, physical discomfort, such as muscle pain and headaches resulting from prolonged smartphone use, can also influence sleep latency (45). Further, a longitudinal study performed in the third and seventh weeks of the COVID-19 lockdown revealed that increased electronic device usage affects the time course of sleep disturbance, which includes prolonged sleep onset latency, reduced sleep duration, decreased sleep quality, and so on (83). Sleep disturbance, especially difficulty initiating sleep and early morning awakening, has long been related to depression in previous studies; however, the direction of this association is still unclear. Insomnia has been regarded as a secondary manifestation, predictive prodromal symptom, or risk factor of depression (48). The current study verified the mediating role of smartphone use duration and sleep disturbance in the association between fear of COVID-19 and depression; however, since it was based on only one wave survey, the directions of these associations need more future follow-up studies based on longitudinal data.

The third pathway identified in the current study was feeling extremely scared → DIS/EMA → depression. The present study showed that people who felt extremely scared of COVID-19 had sleep disturbance and shorter sleep duration. Trauma-induced sleep disruption has been noted to be a precursor to subsequent depression development (84). Similarly, it has been reported that difficulty in sleep onset or maintenance resulting from trauma exposure could influence the outcome of major depression (85). People with difficulty initiating sleep are more likely to develop intrusive thoughts and rumination (86, 87), which may contribute to depression. In addition, the inability to fall asleep can trigger negative schemas, such as helplessness and a lack of control, which may predispose people to depressive symptoms (88). Therefore, interventions focused on improving sleep, particularly on sleep initiation and early morning awakening, may aid in preventing and intervening against depression during pandemics such as COVID-19.

In addition, this study found that having someone infected in the community was also a risk factor for depression. This uncertainty may increase people's worry, panic, and anxiety, reinforcing psychological stress (89–91), which may be a predisposing factor for depression. However, this potential intermediate mechanism deserves further investigation.

## Limitations

The present study had several limitations. First, the sample was limited because it was not strictly random sampling. Second, although it was a national sample, there was only a tiny sample from the worst-affected areas, and there is limited generalizability to other regions. Third, the evaluation in the current study was based on participants' retrospective memory, thus potentially introducing self-reporting and memory bias into the findings. Fourth, the PHQ-9 was initially designed for screening depression within the past 2 weeks; however, we used it to screen depression in the past month, which may have had a potential influence on the validity and reliability of the scale, and further studies are warranted to explore this question. Fifth, pandemic fear, sleep disturbances, and smartphone use were measured by self-designed questions in the current study; therefore, the data could only be seen as explorative data and be interpreted with caution when compared with other data based on different scales. Finally, the current study investigated sleep disturbance during the first 2 months since the COVID-19 outbreak and depression approximately the second month after the outbreak, constituting a temporal relationship. However, since there is only one wave investigation, the directions of the associations should be explained in light of its limitations. Moreover, evidence from a growing number of studies has indicated that COVID-19-related adverse psychological sequelae may persist for months or years (92–94) or come and peak later than the actual pandemic (95). Therefore, future longitudinal follow-up studies are warranted to clarify the trajectories of these variables and their longitudinal associations.

## Conclusion

The current study examined interactions between the COVID-19 exposure severity, daily hours of smartphone use, sleep disturbance, and the prevalence of depression at the early stage of COVID-19 in the general population of China. Results showed that subjectively feeling scared of the pandemic was associated with depression, independent of the effect of age, gender, educational level, vocation, and isolation duration. Meanwhile, feeling scared of the pandemic was also associated with longer smartphone use, shorter night sleep duration, and sleep disturbance. Moreover, smartphone use duration, difficulty initiating sleep, and early morning awakening mediated the association between feeling scared and depressed. The potential impact of various other psychosocial factors—such as economic factors, occupation, age, gender, and home isolation duration—was also examined; however, none of these factors were found to have a significant effect on depression. Psychological intervention during COVID-19 should pay more attention to individuals' subjective feelings. At the same time, smartphone use and sleep disturbance could be potential targets for monitoring and intervention for depression.

Given the critical role of problematic use of smartphones in sleep disturbance and depression, various positive coping strategies (such as exercise, printed book reading, listening to music, yoga, meditation, and so on) should be recommended to the public to reduce screen time. As it may be challenging to evaluate the severity of the negative influence of smartphone addiction and when and how to implement an intervention, the presence of sleep disturbance could be a valuable warning sign; that is, sleep disturbance should be timely and well-intervened in during the time of a pandemic, especially in individuals with excessive smartphone use. Finally, as the mental health impact of the pandemic may vary over time, there was a need for dynamic evaluation of the mental health problem and the delivery of long-term psychosocial care or support to vulnerable populations, such as those feeling extreme fear.

## Data availability statement

The original contributions presented in the study are included in the article/Supplementary material, further inquiries can be directed to the corresponding authors.

## Ethics statement

The study was approved by the Ethics Committee of the Sichuan University. The patients/participants provided their written informed consent to participate in this study.

## Author contributions

WT and CQ: conceptualization and resources. WT and GL: methodology, investigation, data curation, and project administration. WT and HL: software. GL and HL: formal analysis. GL: writing—original draft preparation and funding acquisition. WT: writing—review and editing. CQ: supervision. All authors have read and agreed to the published version of the manuscript.

## Funding

This work was supported by the China Postdoctoral Science Foundation (2018M643488), the National Natural Science Foundation of China (81901928), and the program of the China Scholarship Council (202006240034).

## Conflict of interest

The authors declare that the research was conducted in the absence of any commercial or financial relationships that could be construed as a potential conflict of interest.

## Publisher's note

All claims expressed in this article are solely those of the authors and do not necessarily represent those of their affiliated organizations, or those of the publisher, the editors and the reviewers. Any product that may be evaluated in this article, or claim that may be made by its manufacturer, is not guaranteed or endorsed by the publisher.
