# Supplementary material for: Fear of COVID-19, prolonged smartphone use, sleep disturbances, and depression in the time of COVID-19: A nation-wide survey
Source: Front Psychiatry. 2022 Oct 14;13:971800. doi: 10.3389/fpsyt.2022.971800 (PMC9614217; doi:10.3389/fpsyt.2022.971800)
Supplement: Supplementary file 1 [file Table_1.pdf]

Supplementary Materials (Table S1-S3):

Table S1 Duration of smartphone use in different subgroups.

Variables	*N(%)*	Phone-time Mean(SD)	t/F	P
Total	1,250	3.51(1.45)		
Age				
18-35	450(36.0)	3.80(1.486)[a]	13.948	<0.001
36-55	749(59.9)	3.36(1.414)[b]		
56-72	51(4.1)	3.22(1.316)[b]		
Gender				
Male	454(36.3)	3.83(1.476)	-5.980	<0.001
Female	796(63.7)	3.33(1.405)		
Vocation				
Health workers	65(5.2)	3.23(1.156)[a]	41.974	<0.001
Community health staff	149(11.9)	2.56(1.343)[b]		
Other	1,036(82.9)	3.67(1.429)[c]		
Home isolated (>2weeks)				
Yes	974(77.9)	3.53(1.420)	-0.860	0.390
No	276(22.1)	3.45(1.556)		
Income decrease				
Yes	501(40.1)	3.71(1.560)	3.833	<0.001
No	749(59.9)	3.38(1.359)		
Educational level				
Junior high school and below	254(20.3)	3.54(1.476)[a]	3.575	0.007
Senior high school	335(26.8)	3.29(1.461)[b]		
Junior college	277(22.2)	3.52(1.522)[a,b]		
Bachelor degree	344(27.5)	3.66(1.335)[a]		
Master degree and above	40(3.2)	3.88(1.488)[a]		
Type of exposure				
Felt extremely scared				
Yes	262(21.0)	3.72(1.476)	-2.663	0.009
No	998(79.0)	3.46(1.440)		
Someone in their community was infected				

Yes	19(1.5)	3.51(1.449)	-0.840	0.401
No	1231(98.5)	3.79(1.584)		
A friend or relative was infected				
Yes	56(4.5)	3.86(1.554)	-1.823	0.069
No	1194(95.5)	3.50(1.445)		
Living in the worst-hit areas				
Yes	78(6.2)	3.97(1.450)	-2.915	0.004
No	1172(93.8)	3.48(1.447)		

Note. The p-value is for the difference in daily smartphone use time in different subgroups under the titled demographic and exposure category (for example, subgroups in age, gender, vocation, and so on). The letter "a" "b" or "C" denotes the column proportion does not differ significantly from those with the same superscripts at the 0.05 level but differed significantly from those with different superscripts at the 0.05 level.

Table S2 Average sleep duration per night in different subgroups.

Variables	*N*	Sleep duration	t/F	P
Total	1,250	9.52(1.51)		
Age				
18-35	450(36.0)	7.81(1.56)[a]	16.310	<0.001
36-55	749(59.9)	7.39(1.47)[b]		
56-72	51(4.1)	6.86(1.28)[c]		
Gender				
Male	454(36.3)	7.56(1.47)	-0.712	0.476
Female	796(63.7)	7.50(1.54)		
Vocation				
Health workers(non-community)	65(5.2)	7.16(1.22)[a]	31.186	<0.001
Community health staff	149(11.9)	6.68(1.42)[b]		
Other	1,036(82.9)	7.67(1.50)[c]		
Home isolated (>2weeks)				
Yes	974(77.9)	7.58(1.51)	-2.673	0.008
No	276(22.1)	7.31(1.51)		
Income decrease				
Yes	501(40.1)	7.52(1.57)	-0.066	0.948
No	749(59.9)	7.52(1.48)		
Educational level				
Junior high school or below	254(20.3)	7.65(1.71)	1.729	0.141
Senior high school	335(26.8)	7.35(1.54)		
Junior College	277(22.2)	7.53(1.42)		
Bachelor degree	344(27.5)	7.58(1.40)		
Master degree and above	40(3.2)	7.61(1.45)		
Type of exposure				
Felt extremely scared				
Yes	262(21.0)	7.30(1.80)	2.322	0.021
No	998(79.0)	7.58(1.42)		
Community infected				
Yes	19(1.5)	6.63(1.80)	2.592	0.010
No	1231(98.5)	7.54(1.51)		

Friend or relative infected				
Yes	56(4.5)	7.22(1.89)	1.219	0.228
No	1194(95.5)	7.54(1.49)		
Living in the worst-hit areas				
Yes	78(6.2)	7.39(1.91)	0.635	0.527
No	1172(93.8)	7.53(1.48)		

Note. The p-value is for the difference in sleep duration per night in different subgroups under the titled demographic and exposure category (for example, subgroups in age, gender, vocation, and so on). The letter "a" "b" or "C" denotes the column proportion does not differ significantly from those with the same superscripts at the 0.05 level but differed significantly from those with different superscripts at the 0.05 level.

Table S3. Mediation analyses to assess the direct and indirect effects of exposure to depression, with the smartphone use duration, difficulty initiating sleep, and early morning awakening as mediators

Path	*Effect*	*SE*	*p*	95% CI
Feeling scared(X)→Phone_time(M1)→ DIS(M2) →Depression(Y)				
Total effect of X on Y	3.419	0.370	<0.001	2.693-4.144
Direct effect of X on Y	1.989	0.325	<0.001	1.351-2.627
Indirect effect of X on Y(Total)	1.430	0.209	-	1.052-1.863
Indirect effect of X on Y(Via M1)	0.077	*0.039*	-	*0.018-0.173*
Indirect effect of X on Y(Via M2)	1.317	*0.202*	-	*0.946-1.733*
Indirect effect of X on Y(Via M1→ M2)	0.036	*0.020*	-	*0.008-0.089*
X→M1	0.239	0.101	0.018	0.042-0.437
M1→Y	0.322	*0.085*	<0.001	*0.155-0.489*
X→M2	0.523	*0.073*	<0.001	*0.380-0.666*
M2→Y	2.517	0.159	<0.001	2.204-2.829
M1→M2	0.059	0.019	0.002	0.022-0.097
Feeling scared(X)→Phone_time(M1)→ EMA (M3) →Depression(Y)				
Total effect of X on Y	3.419	0.370	<0.001	2.693-4.144
Direct effect of X on Y	2.097	0.348	<0.001	1.416-2.779
Indirect effect of X on Y(Total)	1.322	0.181	-	0.876-1.591
Indirect effect of X on Y(Via M1)	0.089	0.045	-	0.020-0.203
Indirect effect of X on Y(Via M3)	1.209	0.181	-	0.876-1.591
Indirect effect of X on Y(Via M1→M3)	0.023	0.015	-	0.003-0.065
X→M1	0.239	0.101	0.018	0.042-0.437
M1→Y	0.373	0.087	<0.001	0.202-0.545
X→M3	0.601	0.078	<0.001	0.449-0.754
M3→Y	2.011	0.147	<0.001	1.722-2.299
M1→M3	0.049	0.021	0.019	0.008-.089

Note. Phone_time: daily smartphone use duration; DIS: difficulty initiating sleep; EMA: early morning awakening.
